# Uncommon Neuro-Ophthalmologic Findings in Pediatric Lyme Disease

**DOI:** 10.7759/cureus.101250

**Published:** 2026-01-10

**Authors:** Umme Habiba Fatima Syeda, Nika Bakshi, Eric Sherman

**Affiliations:** 1 Pediatrics, Corewell Health William Beaumont University Hospital, Royal Oak, USA; 2 Medicine, Oakland University William Beaumont School of Medicine, Auburn Hills, USA; 3 Ophthalmology, Corewell Health William Beaumont University Hospital, Royal Oak, USA

**Keywords:** abducens nerve palsy, lyme disease, neuroborreliosis, ocular lyme disease, pediatrics

## Abstract

Lyme disease (LD) is the most prevalent tick-borne illness in the United States and Europe, caused by a spirochete transmitted through the bite of an infected *Ixodes* tick. In this report, we present a case of disseminated Lyme disease in a four-year-old female who presented with constitutional symptoms and a recurrent, migratory erythema migrans-like rash for one month. The subsequent onset of eye deviation and vision complaints prompted referral, which revealed bilateral optic nerve edema and a left abducens nerve (CN VI) palsy. Cerebrospinal fluid analysis demonstrated an elevated Lyme antibody index, pleocytosis, and an elevated intracranial pressure of 40 cm H₂O. These findings are consistent with Lyme neuroborreliosis based on European Federation of Neurological Societies (EFNS) guidelines. The patient was treated with prednisolone eye drops, ceftriaxone, acetazolamide for intracranial hypertension, and doxycycline. Follow-up examination demonstrated clinical resolution of CN VI palsy and papilledema. This case highlights the importance of maintaining a high index of suspicion for Lyme disease in pediatric patients with neurologic or ocular symptoms following systemic or dermatologic findings suggestive of early Lyme disease. The rapid improvement of uncommon ocular manifestations further illustrates the potential role of timely inpatient evaluation and therapy.

## Introduction

Lyme disease (LD) is the most common tick-borne illness in North America and Europe [1]. It is caused by the bacterium *Borrelia burgdorferi*, which is transmitted by certain *Ixodes* ticks that live throughout temperate regions of the Northern Hemisphere [1]. It has become the most commonly reported illness transmitted by vectors in the United States [1]. Children between the ages of 5 and 9-especially boys-are the most commonly affected [2].

The clinical manifestations of LD tend to progress through three stages; however, these stages do not necessarily occur in a sequential manner, and patients, particularly children, may present with disseminated disease without a prolonged or clearly recognized early localized phase [3]. Early disease may be nonspecific and include flu-like symptoms such as fatigue and fever [3]. In the early localized stage, the most recognizable sign is erythema migrans, a red rash with central clearing that appears at the site of the tick bite [3]. The early disseminated stage may involve multiple erythema migrans lesions, Borrelial lymphocytoma, neurological involvement, cardiac issues, or joint pain [3]. In the late disseminated stage, patients may develop acrodermatitis chronica atrophicans, chronic arthritis, or ongoing neurological problems such as encephalomyelitis [4]. Although the clinical stages of Lyme disease are similar in pediatric patients and adults, children may be more likely to present with nonspecific symptoms or delayed recognition of early cutaneous findings, which can complicate diagnosis [2]. Importantly, early disseminated Lyme disease may occur in pediatric patients even in the absence of a recognized tick bite or prolonged diagnostic delay, reflecting nonspecific early symptoms and unreliable exposure history in children [5].

Regarding ocular involvement, LD can affect the eyes at any stage and may involve both eyes. Ocular involvement is considered an uncommon manifestation of LD and is more frequently reported in the setting of disseminated infection [6]. Although ocular involvement may occur at any stage, more severe neuro-ophthalmic manifestations are more commonly associated with disseminated infection [6]. The most common ocular manifestation, typically seen in early disease, is follicular conjunctivitis, which appears in approximately 11% of patients [6]. As the disease progresses, a wider range of eye pathology can develop, such as uveitis, retinal vasculitis, neuroretinitis, episcleritis, keratitis, papillitis, optic neuritis, and even palsies of cranial nerves III, V, VI, and VII [6]. Such manifestations of Lyme disease have been previously reported in both adult and pediatric populations [6].

Together, these varying clinical presentations highlight the diagnostic complexity of Lyme disease in pediatric patients, especially when early manifestations are nonspecific.

## Case presentation

In July 2025, a four-year-old female with no significant past medical history presented to the infectious disease clinic with a one-month history of recurrent annular rashes. The rashes initially appeared on the lower extremities and buttocks following a brief febrile illness that included one day of vomiting. According to her mother, the lesions were large, ovoid, annular, and demonstrated central clearing; one lesion later appeared on the upper arm. Each eruption resolved spontaneously within one to two weeks; however, new lesions continued to develop, including one on the cheek. Associated symptoms included leg pain without swelling or gait changes, recurrent frontal headaches, fatigue, decreased appetite, and reduced activity. There was no travel history or tick bite history.

Initial laboratory evaluation by her pediatrician - including complete blood count, C-reactive protein, and a nasopharyngeal respiratory pathogen panel-was unremarkable (Table 1). The labs also reported normal white blood cell count and mild thrombocytosis, which may be seen in inflammatory or infectious conditions. Erythrocyte sedimentation rate was slightly elevated. Given the spontaneous resolution of individual lesions and initially mild systemic symptoms, the patient was initially suspected to have a viral illness. However, due to the recurrence and migratory nature of the rashes with central clearing and the development of systemic symptoms, concern for early disseminated Lyme disease prompted referral to an infectious disease specialist, where Lyme serology returned positive. Additional workup revealed elevated antistreptolysin O (ASO) and anti-DNase B titers, Epstein-Barr virus IgM positivity, and a positive Group A streptococcus polymerase chain reaction (Table 2).

**Table 1:** Initial laboratory evaluation Abbreviations: MCV: Mean corpuscular volume; MCH: Mean corpuscular hemoglobin; MCHC: Mean corpuscular hemoglobin concentration; RDW: Red cell distribution width; MPV: Mean platelet volume.

Laboratory Test	Result	Reference Range
White Blood Cell Count	8.87 ×10³/µL	5.00–14.00 ×10³/µL
Red Blood Cell Count	4.47 ×10⁶/µL	3.70–5.30 ×10⁶/µL
Hemoglobin	11.5 g/dL	11.0–14.0 g/dL
Hematocrit	36.1%	33.0–42.0%
Mean Corpuscular Volume (MCV)	80.8 fL	70.0–90.0 fL
Mean Corpuscular Hemoglobin (MCH)	25.7 pg	23.0–33.0 pg
Mean Corpuscular Hemoglobin Concentration (MCHC)	31.9 g/dL	32.0–37.0 g/dL
Red Cell Distribution Width (RDW)	12.3%	11.5–14.5%
Platelet Count	586 ×10³/µL	140–440 ×10³/µL
Mean Platelet Volume (MPV)	10.0 fL	9.5–12.2 fL
Neutrophils Absolute Count	4.34 ×10³/µL	1.70–9.00 ×10³/µL
Lymphocytes Absolute	3.46 ×10³/µL	1.50–8.00 ×10³/µL
Monocytes Absolute	0.49 ×10³/µL	0.10–1.00 ×10³/µL
Eosinophils Absolute Count	0.48 ×10³/µL	0.00–0.60 ×10³/µL
Basophils Absolute Count	0.09 ×10³/µL	0.00–0.30 ×10³/µL
Immature Granulocyte Absolute Count	0.01 ×10³/µL	0.00–0.04 ×10³/µL
Immature Granulocyte Automated	0.10%	0.0–1.0%
Nucleated Red Blood Cells Automated	0.0%	≤0.0%
Erythrocyte Sedimentation Rate	28 mm/hr	0–20 mm/hr
C-Reactive Protein	0.50 mg/dL	0.00–0.80 mg/dL
Respiratory Pathogen Panel	Negative	Negative

**Table 2:** Additional laboratory studies

Laboratory Test	Result	Reference Range
Lyme Total Antibody (total antibody with confirmatory IgM and IgG western blot)	Positive (IgM and IgG)	Negative
Antistreptolysin O	460 IU/mL	<100 IU/mL
Anti-DNase B	388 U/mL	0–250 U/mL
Epstein-Barr Virus IgM	Positive	Negative
Group A Streptococcus PCR	Positive	Negative

Although additional infectious markers were identified as part of the broader infectious evaluation, including Epstein-Barr virus IgM positivity and elevated ASO and anti-DNase B titers, these findings were interpreted as either incidental or due to prior or concurrent exposures rather than the cause of the patient’s presentation. The rash, systemic symptoms, and subsequent development of neurologic and ocular manifestations were felt to be most consistent with Lyme disease, which was supported by positive Lyme serology with both IgM and IgG antibodies.

**Figure 1:**
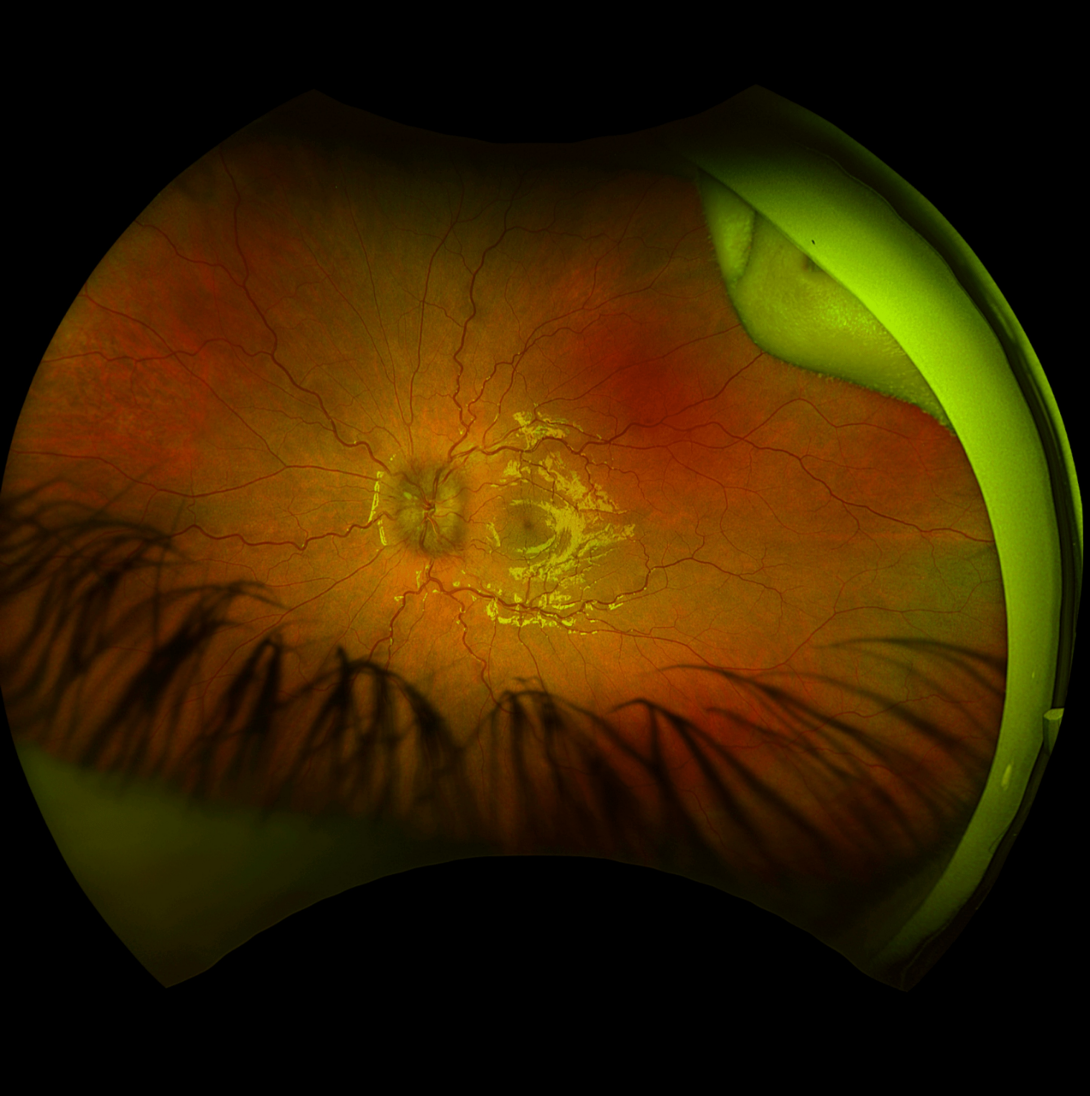
Fundus photograph demonstrating optic disc edema consistent with papilledema in Lyme neuroborreliosis.

She was referred to ophthalmology for complaints of blurred vision several weeks after developing a rash. Additionally, the patient's mother noted inward deviation of her left eye. Detailed ophthalmologic evaluation demonstrated bilateral optic nerve edema and a left CN VI palsy. Fundus photography demonstrated bilateral optic disc edema consistent with papilledema (Figure 1). Subepithelial infiltrates were observed in the cornea of the right eye. She was then transferred to the emergency department for inpatient admission to the pediatric floor for management of suspected disseminated Lyme disease.

On arrival, computed tomography (CT) of the head ruled out an acute process. The white blood cell count, hemoglobin, hematocrit, and complete metabolic panel were all within normal ranges. Blood cultures were obtained, and a lumbar puncture was performed to evaluate for Lyme meningitis. Clinically, the patient did not have classic meningeal signs, such as nuchal rigidity or photophobia, and reported headaches and blurred vision as the primary neurologic symptoms. Cerebrospinal fluid (CSF) analysis revealed an elevated nucleated cell count of 42 cells/mcL (reference range: <5 cells/mcL), elevated protein at 50 mg/dL (reference range: 15-45 mg/dL), and slightly low glucose of 48 mg/dL (reference range: 50-80 mg/dL) (Table 3). The opening pressure was elevated at 40 cm H₂O. These findings-lymphocytic-predominant pleocytosis with moderately elevated protein and near normal glucose-are consistent with aseptic meningitis patterns described in pediatric Lyme neuroborreliosis. Additionally, the meningitis/encephalitis panel was unremarkable, while the CSF Lyme antibody index was elevated at 2.88 IV (reference range: ≤0.90 IV), supportive of Lyme neuroborreliosis in the appropriate clinical context. An elevated CSF Lyme antibody index, in contrast to serum serology alone, suggests intrathecal antibody production and supports central nervous system involvement when interpreted alongside compatible clinical findings. She was started on ceftriaxone 100 mg/kg and admitted to the pediatric floor. Further inpatient evaluation included an electrocardiogram and a brain/orbit MRI, obtained to evaluate the causes of papilledema and cranial nerve VI palsy, which was unremarkable.

**Table 3:** Cerebrospinal fluid analysis Abbreviation: CSF: Cerebrospinal fluid.

Laboratory Test	Result	Reference Range
Total Nucleated Cells	42 cells/µL	≤5 cells/µL
Protein, CSF	50 mg/dL	15–45 mg/dL
Glucose, CSF	48 mg/dL	50–80 mg/dL
Lyme Disease Antibody Index, CSF	2.88 IV	≤0.90 IV

The patient was treated with a one-time dose of intravenous ceftriaxone 100 mg/kg, with plans to transition to 75 mg/kg daily. However, due to a lack of parenchymal central nervous system involvement, she was transitioned from ceftriaxone to oral doxycycline 2.2 mg/kg for 21 days. The patient was also started on acetazolamide 15 mg/kg two times daily for elevated intracranial pressure. She was also prescribed prednisolone acetate 1% once daily by ophthalmology for subepithelial infiltrates.

She demonstrated clinical improvement at follow-up over the following two months. Optic disc edema had resolved, extraocular movements were full, and the subepithelial corneal infiltrates had also resolved. At this time, acetazolamide was stopped.

## Discussion

Pediatric Lyme disease is the most prevalent vector-borne illness in the United States and Europe, with children ages 5-9 disproportionately affected, likely reflecting increased outdoor exposure and limited tick-avoidance in this age group [1,2]. Early disseminated disease can occur within weeks to months after a tick bite, which can range from neurologic manifestations to cutaneous findings [7]. Our case demonstrates that severe neuroborreliosis can occur in children even in the absence of a tick bite or travel history, often leading to diagnostic delay.

Our patient’s presentation was initially misclassified as a viral exanthem not only due to the absence of a known tick bite or travel history, but also due to the spontaneous resolution of lesions and initially mild systemic symptoms. This diagnostic challenge resulted in weeks of outpatient observation during which dissemination of the disease occurred. Delayed initiation of antimicrobial therapy likely permitted hematogenous dissemination and central nervous system involvement, as prompt treatment of Lyme disease is known to interrupt disease progression [4]. Such presentations highlight a challenge in pediatric Lyme disease, in which the absence of epidemiologic exposure, combined with transient symptom resolution and mild early manifestations, may obscure evolving infection [5]. According to some studies, only 18.5% of children with LD have a recognized tick bite [5]. Our patient was eventually referred to an infectious disease specialist following the recurrence of erythema migrans rashes. During this referral, the patient had already complained of vision impairment. This case illustrates established clinical principles that erythema migrans and evolving symptoms remain central to the diagnosis of Lyme disease, even in the absence of known tick exposure or persistent systemic symptoms, particularly in pediatric patients.

When Lyme disease goes unrecognized, dissemination to the nervous system can occur, resulting in Lyme neuroborreliosis [4,7]. Lyme neuroborreliosis most commonly presents with neurologic manifestations, including cranial neuropathies, lymphocytic meningitis, and, less often, neuro-ophthalmologic involvement [4]. In this case, an untreated infection was followed by the development of neuro-ophthalmologic pathology weeks after the initial infection. Ocular involvement in LD is well-documented but uncommon, with most cases presenting with milder manifestations such as follicular conjunctivitis [8]. Conversely, motility disorders such as abducens nerve palsy are unusual, since facial nerve palsy accounts for the majority of Lyme disease cranial neuropathies [8,9]. The combination of papilledema and CN VI palsy seen in our patient has been reported infrequently and reflects elevated intracranial pressure. This presentation is consistent with a rare but recognized phenotype of secondary intracranial hypertension (pseudotumor cerebri-like syndrome) associated with Lyme neuroborreliosis [10]. Pediatric case reports and small series describe this phenotype with visual outcomes ranging from full recovery to permanent visual impairment when intracranial hypertension is prolonged [10,11].

This case adds to the existing pediatric Lyme disease literature by illustrating an uncommon neuro-ophthalmologic presentation in the setting of early disseminated infection. It highlights that Lyme-associated papilledema or ocular motility issues may serve as early clinical indicators of central nervous system involvement, even in the absence of epidemiologic factors. Moreover, the patient’s favorable visual outcome aligns with outcomes previously described in pediatric cases of Lyme neuroborreliosis managed with timely inpatient therapy, underscoring the potential for neurologic recovery even in severe or atypical presentations [11].

## Conclusions

This case underscores the importance of prompt recognition and evaluation when pediatric Lyme disease progresses to neuroborreliosis, especially in the presence of visual symptoms. Ocular manifestations in Lyme disease typically consist of mild presentations, making the neuro-ophthalmologic findings seen in this case an indicator of central nervous system involvement, even in the absence of clear epidemiologic exposure. The patient experienced clinical improvement following treatment of the infection with antimicrobial therapy and acute management of intracranial hypertension with acetazolamide. This outcome is consistent with management strategies described in the existing literature for Lyme neuroborreliosis and its complications, while illustrating the potential recovery in severe or atypical pediatric presentations.

## References

[1] Mead P (2022). Epidemiology of Lyme disease. Infect Dis Clin North Am.

[2] Shafquat M, Angulo FJ, Pilz A, Moïsi JC, Stark JH (2023). The incidence of Lyme borreliosis among children. Pediatr Infect Dis J.

[3] Skar GL, Blum MA, Simonsen KA (2025). Lyme Disease. https://pubmed.ncbi.nlm.nih.gov/28613720/.

[4] Cardenas-de la Garza JA, De la Cruz-Valadez E, Ocampo-Candiani J, Welsh O (2019). Clinical spectrum of Lyme disease. Eur J Clin Microbiol Infect Dis.

[5] Nigrovic LE, Neville DN, Balamuth F, Bennett JE, Levas MN, Garro AC (2019). A minority of children diagnosed with Lyme disease recall a preceding tick bite. Ticks Tick Borne Dis.

[6] DesLauriers A, Larochelle M (2025). Lyme disease-associated uveitis: A case report and review emphasizing the importance of travel history and geographic considerations. Moran CORE.

[7] Hu L (2025). Clinical manifestations of Lyme disease in adults. UpToDate.

[8] Balcer LJ, Winterkorn JM, Galetta SL (1997). Neuro-ophthalmic manifestations of Lyme disease. J Neuroophthalmol.

[9] Marques A, Okpali G, Liepshutz K, Ortega-Villa AM (2022). Characteristics and outcome of facial nerve palsy from Lyme neuroborreliosis in the United States. Ann Clin Transl Neurol.

[10] Mah JM, Lo C, O'Connor MD (2024). Isolated intracranial hypertension as a presentation of pediatric Lyme borreliosis: A case report and literature review. Pediatr Neurol.

[11] Rothermel H, Hedges TR 3rd, Steere AC (2001). Optic neuropathy in children with Lyme disease. Pediatrics.

